# A Simple Interface for 3D Position Estimation of a Mobile Robot with Single Camera

**DOI:** 10.3390/s16040435

**Published:** 2016-03-25

**Authors:** Chun-Tang Chao, Ming-Hsuan Chung, Juing-Shian Chiou, Chi-Jo Wang

**Affiliations:** Department of Electrical Engineering, Southern Taiwan University of Science and Technology, 1, Nan-Tai St., Yongkang District, Tainan 71005, Taiwan; tang@stust.edu.tw (C.-T.C.); ma329201@stust.edu.tw (M.-H.C.); chijo@stust.edu.tw (C.-J.W.)

**Keywords:** arduino, robot interface, single camera, 3D position

## Abstract

In recent years, there has been an increase in the number of mobile robots controlled by a smart phone or tablet. This paper proposes a visual control interface for a mobile robot with a single camera to easily control the robot actions and estimate the 3D position of a target. In this proposal, the mobile robot employed an Arduino Yun as the core processor and was remote-controlled by a tablet with an Android operating system. In addition, the robot was fitted with a three-axis robotic arm for grasping. Both the real-time control signal and video transmission are transmitted via Wi-Fi. We show that with a properly calibrated camera and the proposed prototype procedures, the users can click on a desired position or object on the touchscreen and estimate its 3D coordinates in the real world by simple analytic geometry instead of a complicated algorithm. The results of the measurement verification demonstrates that this approach has great potential for mobile robots.

## 1. Introduction

Robot technology has been successfully applied in many areas and is noted for its low cost, greater accuracy and reliability, suitability when there is a labor shortage, and its ability to provide entertainment. In particular robots can execute 3D (dirty, dangerous, and demeaning (or dull, difficult)) jobs for people. Compared with other modern autonomous robots, remote-controlled robots are much safer, which is a major concern in practical applications. Furthermore, increasingly more smartphone-controlled innovations are becoming available partly due to the adoption of smart phones as an everyday device by many people in highly-industrialized societies. However, controlling a mobile robot with a robotic arm by a smartphone or tablet involves a lot of motion actions which involve the visual control interface. This has caused many problems for engineers in the past. In order to improve the practicability of the mobile robot, real-time video transmission function should be added to the design, which allows the robot to operate out of sight of the user. Another crucial issue or function is the 3D position estimation of a target by a single camera. This paper is motivated by the issues mentioned above and aims to find applicable solutions for 3D position estimation with a mobile robot.

The Arduino Yun [1], a microprocessor board with a built-in Wi-Fi module, was utilized to implement the proposed design. Arduino is an open-source electronics platform based on easy-to-use hardware and software launched in 2005. The goal of Arduino is to create easy-to-use and inexpensive control devices for projects [2,3], which is consistent with the requirements of the proposed system.

Radio frequency (RF) wireless remote control is commonly used in commercially available products. Although lately there have been many debates over low-power Wi-Fi and RF (including ZigBee, 6LoWPAN, *etc*.) networks in the context of Internet of Things (IoT) applications and sensor devices [4], since Wi-Fi is available on almost all smartphones, it is adopted as the wireless communication for the control commands and real-time video transmission in the proposed system.

Reconstructing a target’s 3D location presents several problems. For example, an autonomous robot generally uses stereo cameras and range sensors [5,6], but these devices are not commonly used in ordinary remote-controlled mobile robots. Over the past decade, many tracking methods, such as Kalman filters [7], mean-shift [8], and Kanade-Lucas-Tomasi (KLT) algorithms [9] have been developed in computer vision. However, these tracking algorithms are mostly based on gradient changes or object features, which all require complicated computation or prior knowledge of the target objects. Karungaru *et al.* [10] applied a KLT tracker to obtain the distance to the target point for a mobile robot equipped with single camera using motion stereo vision, but the tracking of the feature points by optical flow analysis sometimes failed, further increasing the complexity of the approach. Another approach used a simple humanoid localization with a single camera [11]. With this approach, only simple analytic geometry was applied to determine the real-world coordinates of the robot using the goal posts in a soccer field as a landmark, making it practical and desirable for our system. However, in our application, no object can be used as a landmark.

In this paper, we desire to develop a feasible approach to estimate the target’s 3D position for a mobile robot with a single camera. The camera needs to be properly calibrated and the odometer movement of the robot needs to be well designed to provide motion stereo vision. With the help of a touchscreen and a user-friendly visual control interface, the 3D position calculation will be implemented by simple analytic geometry and triangulation. This paper provides a practical reference for researchers attempting to design a visual remote-controlled system for a mobile robot. The rest of the paper is organized as follows: Section 2 presents the mobile robot design and the visual control interface. In Section 3, the steps for the target’s 3D position estimation is provided and verified. Finally, Section 4 offers brief concluding comments.

## 2. The Mobile Robot Design and the Visual Control Interface

**Figure 1:**
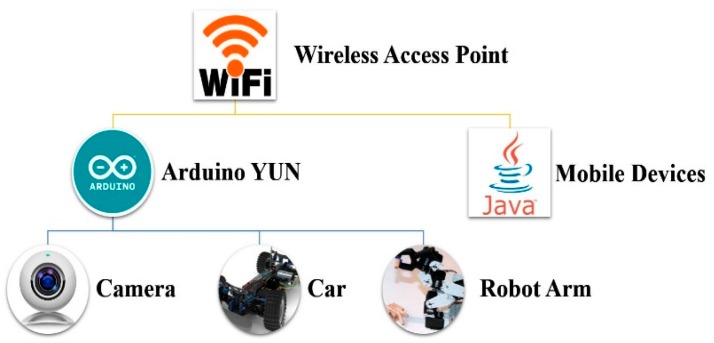
The robotic remote control system architecture.

The proposed robotic remote control system is shown in Figure 1. Wi-Fi is used as the wireless communication because of its prevalence in many devices, such as personal computers, video-game consoles, and smartphones. The Arduino Yun board, based on the ATmega32u4 and the Atheros AR9331, is employed as the mobile robot controller. The board has built-in Ethernet and Wi-Fi support, a USB-A port, a micro-SD card slot, and a micro USB connection. The proposed mobile robot operates as a mobile Wi-Fi hotspot, which is specfically for the remote controller, and achieves smooth communication and control. The software installation or system configuration can be finished wirelessly as the Arduino Yun runs Linino (a MIPS GNU/Linux based on OpenWRT) with the Atheros AR9331 [1].

**Figure 2:**
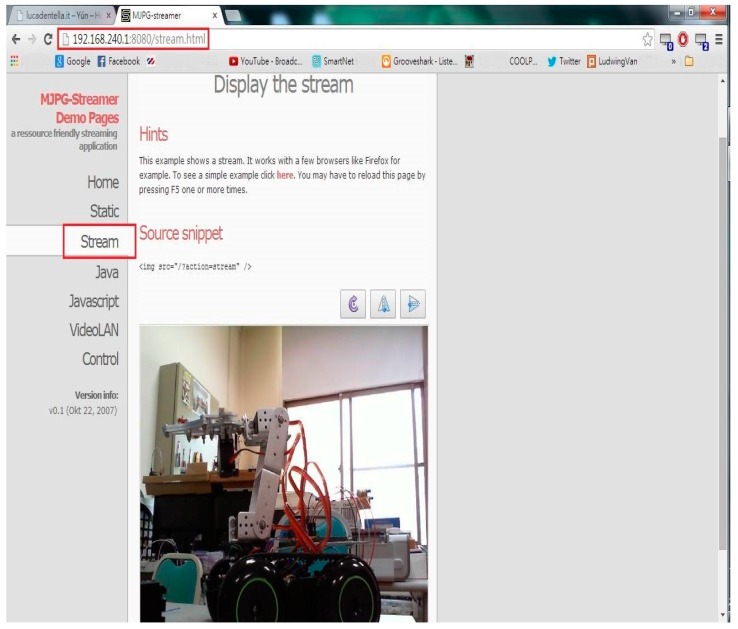
Network video from webcam connected to Arduino Yun.

The Microsoft LifeCam HD-3000 Webcam with a USB interface is connected to the mobile robot for real-time video transmission. Software packages, such as the USB Video Class (UVC) driver, need to be installed on the Arduino Yun. Finally, the PuTTY terminal emulator is applied, which supports SSH (Secure Shell), a terminal protocol for a secure connection between the computer and the Yun [12]. Figure 2 shows the network video taken from the webcam connected to the Arduino Yun, where 192.168.240 is the Yun IP and 8080 is the port number.

**Figure 3:**
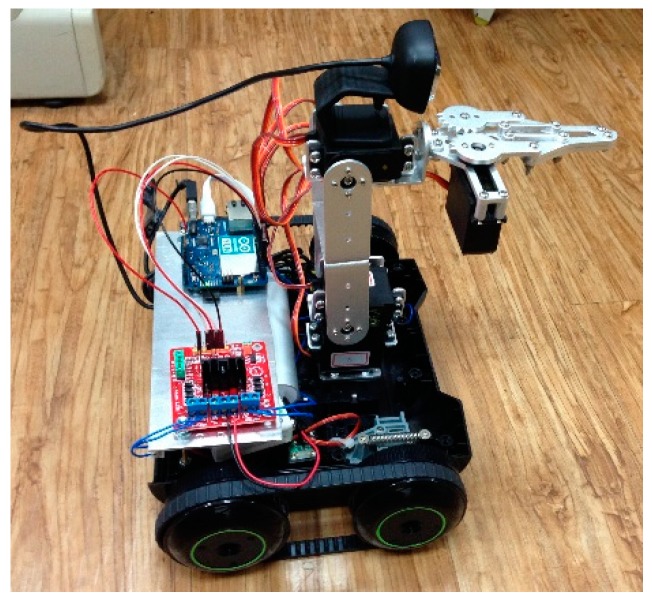
The mobile robot.

Figure 3 shows the completed mobile car. A total of four DC servo motors are used for the robot arm. The bottom one (Motor A) is for base rotation and the top one (Motor D) is for the opening and closing of the claw. The middle two servo motors (Motors B and C) are for the stretch and retraction movement of the robotic arm.

**Figure 4:**
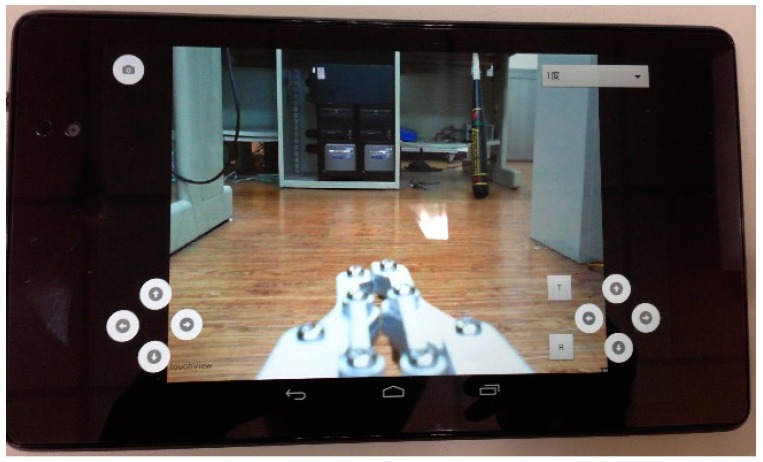
The control interface design.

A user-friendly control interface is important for manipulating the mobile robot. Figure 4 shows the visual control interface design with four buttons in the bottom left for the directional movement of the robot. The buttons in the bottom right control the robotic arm: the “T” and “R” buttons are for Motor D which controls the clamping and releasing ; the “right” and “left” buttons are for Motor A which controls base rotation; and the “forward” and “backward” buttons are for Motors B and C, which balance the claw and perform the claw’s stretch and retraction movement. Moreover, the top left button is for capturing video frame, which is stored in an SD card. Finally, with each click of all the bottom right buttons by the user, the Arduino Yun will send out PWM signals to control the corresponding servo motor to rotate at a fixed angle. The user can click the top right button and a drop-down menu pops out with 1°, 5°, 10°, 20°, or 50° of movement which is to set the fixed rotation angle. The video clips showing how the mobile robot grabs an object have been uploaded to YouTube [13] for the readers’ reference.

## 3. The Target’s 3D Position Estimation

In this section, we show how to estimate the target’s 3D position with motion stereo vision. Firstly, the proposed flow of the robot’s movement and calculation is described. Following this, a practical estimation is conducted to verify the proposed approach.

### 3.1. The Proposed Flow of Robot Movement and Calculation

**Figure 5:**
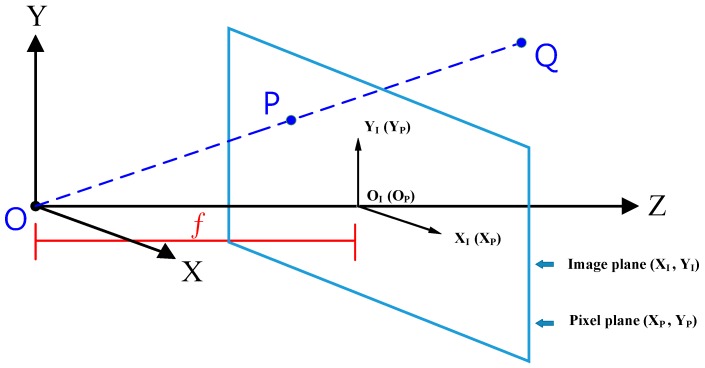
Image projection in relation to the coordinates.

First, the static mapping between the 3D real-world coordinates and the 2D image plane is shown in Figure 5, where *f* is the focal length and the pinhole camera is assumed. There are three coordinate systems in Figure 5, and each has its coordinate origin.

The projection between the target object *Q* (*Q_X_*, *Q_Y_*, *Q_Z_*) in the real world and the *P* point (*P_X_*, *P_Y_*) in the image can be derived by simple triangulation [14], as shown in Equation (1). It is worth mentioning that the *P* point has the same coordinate values in direction *X* and *Y* for both real world coordinates and image plane coordinates, that is, $P_{X}=P_{X_{I}}$ and $P_{Y}=P_{Y_{I}}$.

(1) $$Q_{X}=Q_{z}\frac{P_{X}}{f}\;\text{and}\;Q_{Y}=Q_{Z}\frac{P_{Y}}{f}$$

Equation (1) indicates the importance of *Q_z_*, the distance in *Z* direction. If we can obtain the coordinates of the *P* point in the image plane, we can get all of the coordinates of the *Q* point through *Q_z_*. However, as it is well known, it is hard to obtain the distance to an object just by a single camera. One more transformation is also needed between the image plane coordinates and the pixel plane due to camera pixel specification , as shown in Equation (2):

(2) $$X_{P}=\alpha X_{I}\;\text{and}\;Y_{P}=\alpha Y_{I}$$

where *α* is defined as the pixel number in 1 cm.

**Figure 6:**
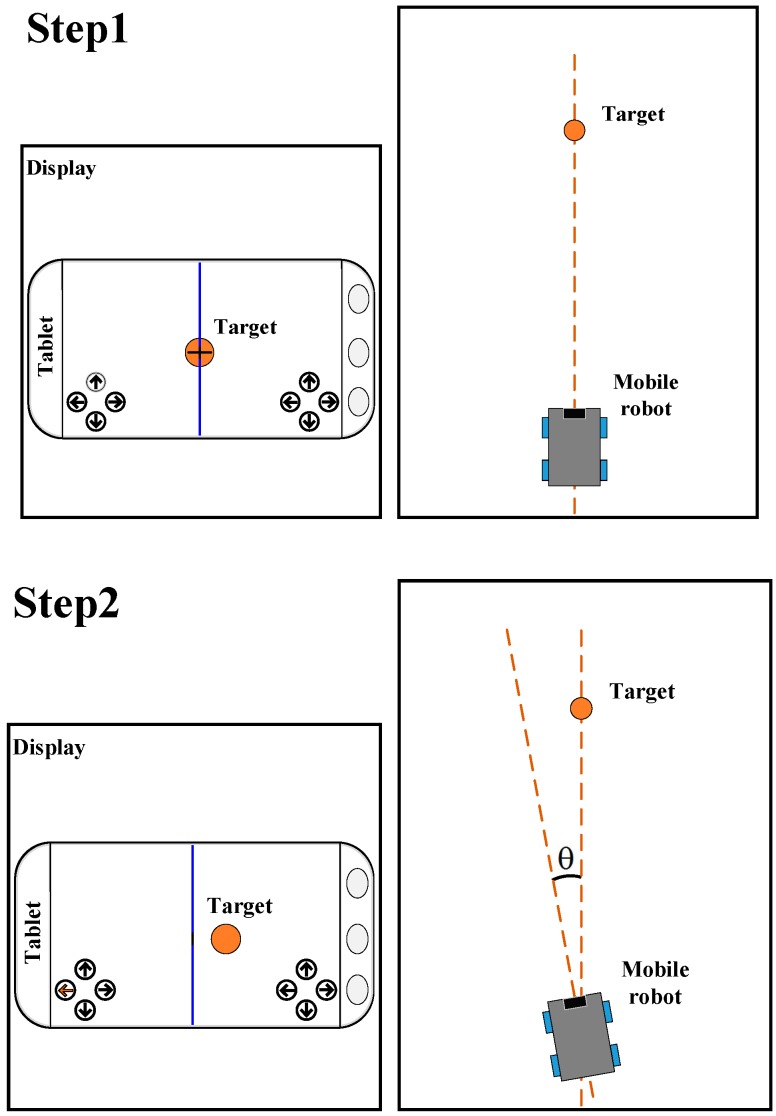

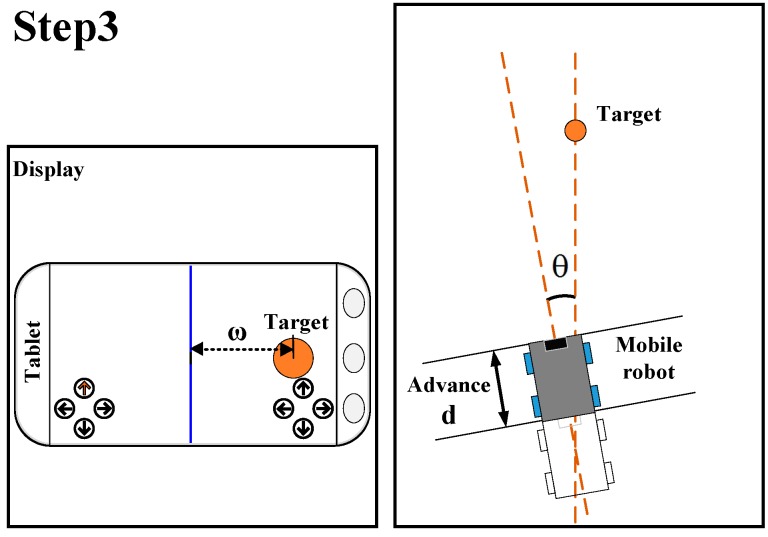
Flow of robot movement.

To estimate the distance of a static object from a single camera, we need to conversely move the robot to show the distance information on the image plane, or the visual interface. Figure 6 shows the proposed flow of robot movement in order to obtain the motion stereo vision for the target’s 3D position. The user’s touchscreen is designed to have a vertical line in the middle of the screen. The proposed steps are outlined as follows:

*Step 1:* The user has to control the robot to face the target, that is, the center of the target should be located on the vertical line on the touchscreen. Then the user must click on the target and the system will check and record this point. Once the check has passed, the process will be successfully activated.

*Step 2:* The robot automatically rotates *θ* degree to the left, and the target will move to the right on the tablet screen. 

*Step 3:* The robot automatically advances forward towards distance *d*, and the target will move to the right on the tablet screen. The user needs to click on the target on the screen again to end the process, and the target’s 3D coordinates will be estimated.

**Figure 7:**
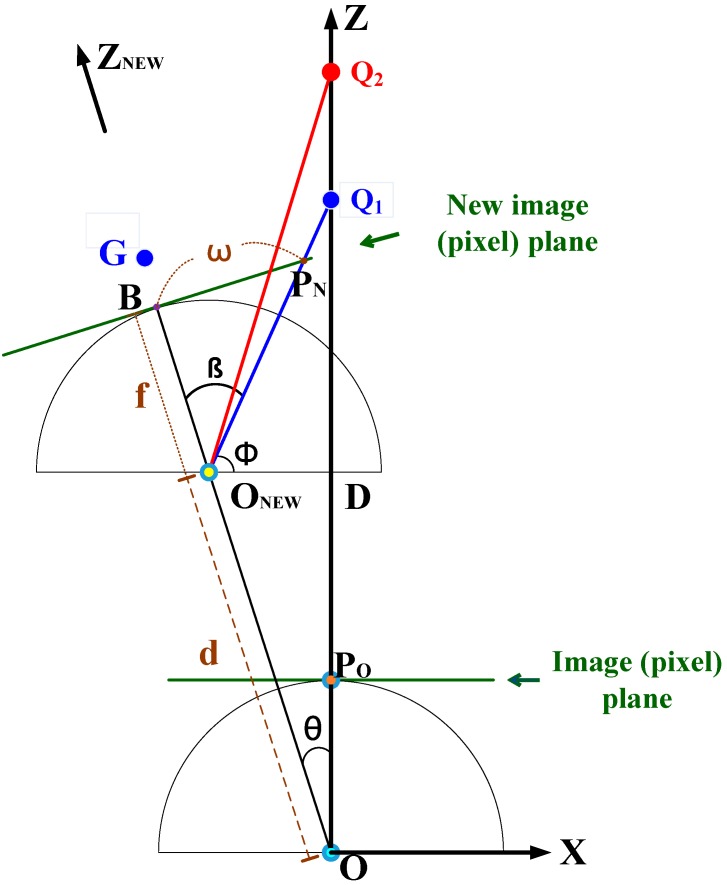
The top view depiction of robot movement.

Figure 7 clearly depicts the robot movement viewed from above. The starting point *O* is the projection center of the camera in the mobile robot. In Step 2, the robot rotates *θ* degrees to the left, and the target points *Q_1_* and *Q_2_* will project to the same position with respect to coordinate *X*. Only when the robot continues to advance towards the target in Step 3 will the target points *Q_1_* and *Q_2_* separate on the image plane in the *X* direction. Let us take target *Q_1_* for example, where *w* is the distance to the middle of the new image plane. The *w* value contains the distance information for targets in the *Z* direction.

Firstly, the distance from starting point *O* to *Q_1_* can be calculated by:

(3) $$\bar{OQ_{1}}=\bar{OD}+\bar{DQ_{1}}=d\;\text{cos}\;\theta +\bar{DQ_{1}}$$

The distance $\bar{DQ_{1}}$ can be calculated by:

(4) $$\bar{DQ_{1}}=\bar{O_{NEW}D}\;\text{tan}\;\phi =d\;\text{sin}\;\theta \;\text{tan}\;\phi$$

Moreover, angle *φ* can be calculated by:

(5) $$\phi =\pi -\beta -(\frac{\pi }{2}-\theta )=\frac{\pi }{2}-\beta +\theta$$

Finally, we can finish the calculation by obtaining angle *β* through the following equation:

(6) $$\beta ={\text{tan}}^{-1}\frac{w}{f}$$

Based on the above derivation, it will be easier to obtain the 3D position of target *Q_1_* with respect to origins *O* or *O_NEW_*. As shown in Figure 7, *P_O_* and *P_N_* are the projected points of *Q_1_* in the original pixel plane and the new pixel plane, respectively. The respective coordinates ($P_{O(X_{P})}$, $P_{O(Y_{P})}$), where $P_{O(X_{P})}≅0$, and ($P_{N(X_{P})}$, $P_{N(Y_{P})}$) are recorded during the user’s two clicks in Step 1 and Step 3. Then, the real-world coordinate of *Q_1_* with respect to origin *O* will be:

(7) $$Q_{1\;\left(w.r.t\;O\right)}=(0,\;\bar{OQ_{1}}\frac{P_{O(Y_{P})}}{f\alpha },\;\bar{OQ_{1}})$$

where the *Y* coordinate value is obtained by Equations (1) and (2), as shown below:

(8) $$Q_{1(Y)\;\left(w.r.t\;O\right)}=Q_{1(Z)}\frac{P_{O(Y)}}{f}=\bar{OQ_{1}}\frac{P_{O(Y_{I})}}{f}=\bar{OQ_{1}}\frac{P_{O(Y_{P})}}{f\alpha }$$

We can also get the real-world coordinate of *Q_1_* with respect to origin *O_NEW_*. First, the *Z* direction will be changed as *Z_NEW_* direction, as shown in Figure 7. The distance of *Q_1_* in *Z_NEW_* direction can be calculated by:

(9) $$\bar{O_{NEW}G}=\bar{OG}-d=\bar{OQ_{1}}\text{cos}\;\theta -d$$

Then by applying Equations (1) and (2), we can get the real-world coordinate of *Q_1_* with respect to origin *O_NEW_* as in the following equation:

(10) $$Q_{1\;\left(w.r.t\;O_{NEW}\right)}=(\bar{O_{NEW}G}\frac{P_{N(X_{P})}}{f\alpha },\;\bar{O_{NEW}G}\frac{P_{N(Y_{P})}}{f\alpha },\;\bar{O_{NEW}G})$$

Finally, let us note that for $Q_{1\;\left(w.r.t\;O\right)}$ and $Q_{1\;\left(w.r.t\;O_{NEW}\right)}$, their respective *Y* coordinate values which represent the height of the target, should be the same. So we have the following equation by Equations (7) and (10):

(11) $$\bar{OQ_{1}}\frac{P_{O(Y_{P})}}{f\alpha }=\bar{O_{NEW}G}\frac{P_{N(Y_{P})}}{f\alpha }$$

### 3.2. Measurement Verification

**Figure 8:**
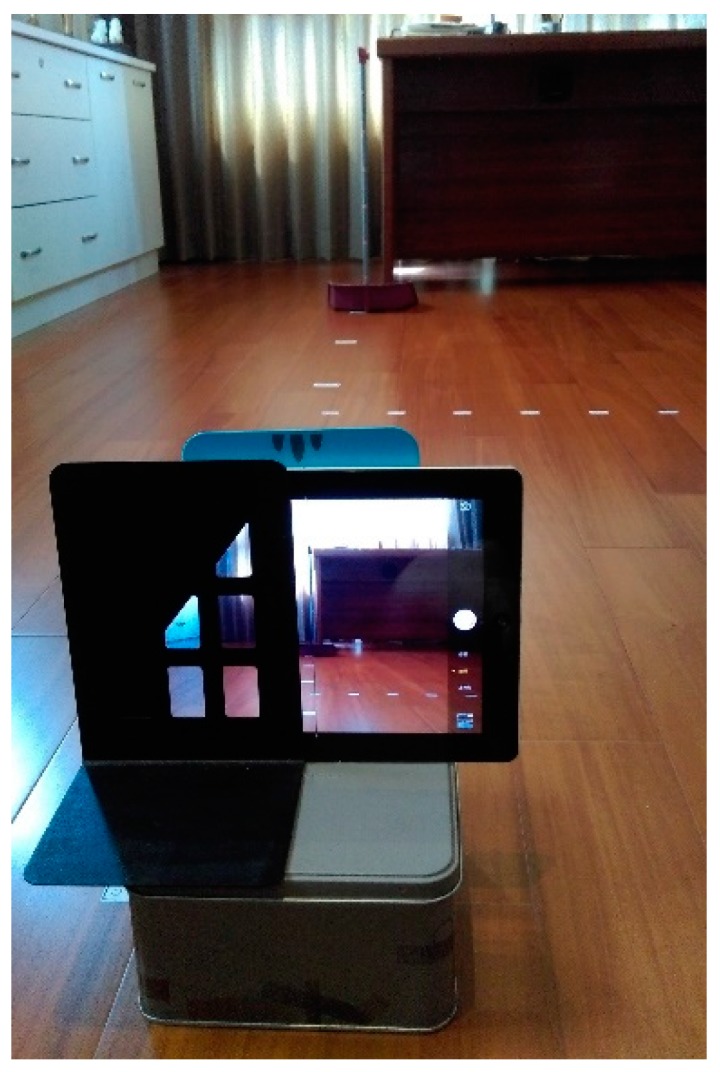
Measurement verification by tablet.

To verify the proposed prototype method and the derived calculation, a tablet with a 9.7 inch display and a 960 × 720 pixels camera was applied as the visual control interface. Figure 8 shows the tablet on top of a metal box which was sandwiched between two bookshelves. Using a long ruler and protractor, we moved the box to simulate the movement and rotation of the mobile robot.

**Figure 9:**
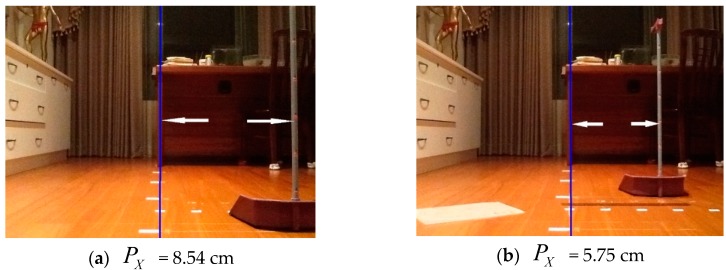

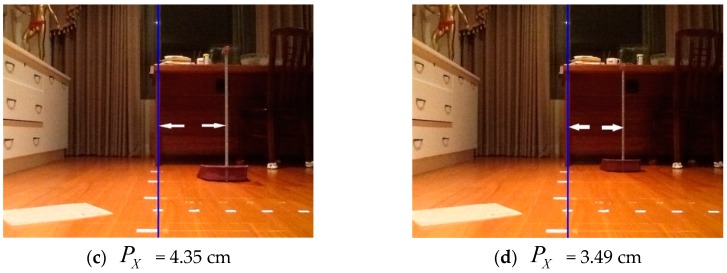
Focal length *f* measurement (*Q_X_* = 36 cm). (**a**) *P_X_* = 8.54 cm; (**b**) *P_X_* = 5.75 cm; (**c**) *P_X_* = 4.35 cm; (**d**) *P_X_* = 3.49 cm.

First, the parameter *α* is about 48.7 pixel/cm according to the tablet screen size and the pixel specification (960 × 720 pixels) of the camera. Furthermore, the measurement of focal length *f* is necessary for a calibrated camera system. In our measurement, a room was plastered with stickers at different distances on the floor, with a movable vertical pole labelled with different heights. Without loss of generality, we will focus on $Q_{X}=Q_{z}\frac{P_{X}}{f}$ in Equation (1) to estimate focal length *f*, so *f* can be calculated by $f=Q_{z}\frac{P_{X}}{Q_{X}}$. In Figure 9, the vertical pole was put at distances ranging from near to far away, with *Q_z_* ranging from 100 cm to 250 cm, but *Q_X_* was kept at 36 cm. The distance between the white double arrow in each picture indicates $P_{X_{P}}$ (pixel) or *P_X_* (cm). All of the data for the four different cases is shown in Table 1. The average *f* was calculated as 24.03, which is adopted in the subsequent calculations.

**Figure 10:**
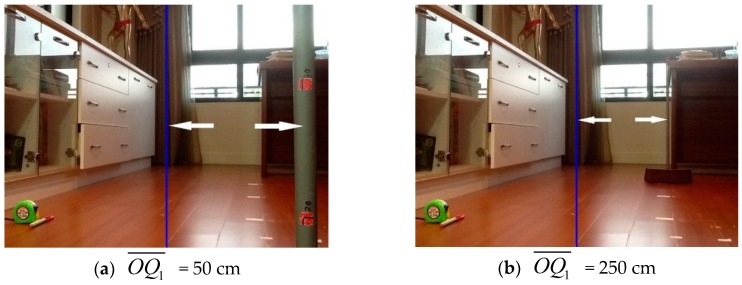
Measurement of distance to the target. (**a**) $\bar{OQ_{1}}$ = 50 cm; (**b**) $\bar{OQ_{1}}$ = 250 cm.

Next, we will verify the proposed flow for estimating the 3D position of a target object. The *θ* is set to 12° and *d* is 20 cm. For simplicity, we will calculate $\bar{OQ_{1}}$ in Equation (3) for the following verification. For the first step in the proposed process, we put the vertical pole in the middle of the screen. Then in the Step 2, the robot was rotated 12° to the left and the vertical pole moved to the right on the screen. Finally, as the robot advanced 20 cm in Step 3, the vertical pole moved to the right again. Figure 10 shows two extreme cases for the target distance measurement after Step 3 was finished, where $\bar{OQ_{1}}$ equals 50 cm and 250 cm, which corresponds to the nearest and farthest cases, respectively. The distance between the white double arrows in each picture indicates $P_{N(X_{P})}$ (pixel) or *w* (cm).

Using the derived equations from the last subsection, the step by step procedure of $\bar{OQ_{1}}$ calculation for the target’s 3D estimation algorithm can be summarized as:

- Given constant values: *θ*, *d*, *α*, *f*.
- Get the coordinates ($P_{O(X_{P})}$, $P_{O(Y_{P})}$) and ($P_{N(X_{P})}$, $P_{N(Y_{P})}$) at the start (Step 1) and end (Step 3) of the proposed approach. The variable *w* equals to $\frac{P_{N(X_{P})}}{\alpha }$.
- Calculate *β* by Equation (6).
- Calculate *φ* by Equation (5).
- Calculate $\bar{DQ_{1}}$ by Equation (4).
- Calculate $\bar{OQ_{1}}$ by Equation (3).
- Estimation for the 3D coordinates of target *Q_1_* with respect to origins *O* or *O_NEW_* can be accomplished by Equations (7) and (10).

Table 2 shows the input $P_{N(X_{P})}$ and the resulting parameters and estimated $\bar{OQ_{1}}$ for the two cases in Figure 10. It seems that the estimation result meets the expectation that the farther away the target is, the larger the error will be, which may be true for any 2D image method used in 3D distance estimation.

Let us review Figure 6 and Figure 7 again, which show the whole process and mathematical derivation of the target’s 3D position estimation. To make the process work, “the robot automatically rotates *θ* degree to the left” in Step 2 is an important step. However, the key point in Step 2 is that the projection center of the camera, the *O* point in Figure 7, must be kept at the same point after the rotation of the mobile robot. As mentioned earlier, we can check this by observing target points at different distances, like *Q_1_* and *Q_2_* in Figure 7, and these should project to the points on the screen with the same *X* coordinate value. But in practice, it may be hard to drive a mobile robot to achieve this. So, in practice, Step 2 and Step 3 can be combined to first obtain the final correct position of the mobile robot by manual operation. After the final ideal position of the robot is obtained, we can then begin designing a method to make the mobile robot reach the correct position automatically.

To implement the automatic process for target’s 3D position estimation, in addition to the above points for Step 2 and Step 3, we should also reconsider the hardware design of the mobile robot. For example, to improve the accuracy, the DC motor which controls the wheels may need to be replaced by a stepping motor. Furthermore, the odometer data often includes some errors due to tire slips, so high friction tires may be necessary.

A remote-controlled mobile robot with video transmission is practical and effective when the operator is unable to see the environment or visibility is poor, but when the mobile robot is fitted with a robotic arm, it could be difficult and time-consuming for an inexperienced user to manipulate the robotic arm to grasp an object. With the proposed target’s 3D position estimation, the user can activate the automatic process when approaching the target, then the mobile robot and robotic arm may reach the appropriate position by inverse dynamics in a short time automatically. Therefore, it has functional applications for robotic grasping tasks, such as obstacle removal or bomb disposal.

For the proposed Wi-Fi mobile robot design in Section 2, it appears that communication is smooth when using the video transmission and control commands. However, when the target’s 3D position estimation in this section is added, the computational consumption may cause a time delay between the click on the touchscreen and the motor reaction. Fortunately, the proposed three steps for the target’s 3D position estimation is activated by the user, and then executed by the system in an automatic way. Thus, the interactive design can be incorporated to reduce the effect of the time delay. Finally, the pitch angle of the camera will affect the positioning performance; thus, the camera and the robotic arm should be reset to the standard position once the target’s 3D position estimation is activated to avoid any error caused by the pitch angle of the camera.

## 4. Conclusions

In this paper, a visual control interface for a mobile robot was proposed, and a simple prototype method for estimating the 3D position of a target was derived and verified. Wi-Fi technology was applied to provide real-time video transmission, which enables the robot to operate in different environments or areas where visibility is poor, limited, or the object is out of sight. It was found that, in addition to the basic movements of the robot, more control actions are needed for the manipulation of the robotic arm and a user-friendly interface is necessary for human operators. In the proposed visual control interface, there are 12 buttons in all for controlling two DC motors and four DC servo motors. Among these buttons, one special button controls a drop-down menu which allows the user to select the degree of movement which allows for a more flexible way to control the servo motor.

The authors have uploaded the operation videos to YouTube for the readers’ reference. The videos show how a skilled user operates the mobile robot to grasp an object with a three-axis robotic arm. However, this will not always be an easy task for an amateur or in an emergency situation. Thus, it will be helpful if we can estimate the 3D position of the desired target, then activate the corresponding automatic process to finish the job by robotic arm inverse dynamics. Although stereo cameras and range sensors can be applied in 3D position reconstruction, we have shown that a simple approach for the target’s 3D estimation with a single, ordinary camera is still a worthwhile research goal. Instead of a complicated theory, this paper proposes a prototype method for estimating the target’s 3D position by simple geometry and triangulation. The proposed steps have been clearly verified and, in future, we hope to make the robot motion and 3D position estimation more accurate.

## Figures and Tables

**Table 1:** Results of focal length *f* measurement (*Q_X_* = 36 cm).

$P_{X_{P}}$ (pixel)	*P_X_* (cm)	*Q_Z_* (cm)	*f* (cm)
416	8.54	100	23.72
280	5.75	150	23.96
212	4.35	200	24.17
170	3.49	250	24.27

**Table 2:** Estimation results of distance to the target.

	$P_{N(X_{P})}$ (Pixel)	*w* (cm)	*β* (In Degree)	*φ* (In Degree)	True $\bar{OQ_{1}}$ (cm)	Estimated $\bar{OQ_{1}}$ (cm)
Case 1	414	8.50	19.48	82.52	50	51.23
Case 2	270	5.54	12.98	89.02	250	262.05

## References

[1] Arduino Yun. https://www.arduino.cc/en/Main/ArduinoBoardYun.

[2] Palma D., Agudo J.E., Sánchez H., Macías M.M. (2014). An Internet of Things example: Classrooms access control over near field communication. Sensors.

[3] Chao C.T., Wang C.W., Chiou J.S., Wang C.J. (2015). An arduino-based resonant cradle design with infant cries recognition. Sensors.

[4] Chen Y., Han F., Yang Y.H., Ma H., Han Y., Jiang C., Lai H.Q., Claffey D., Safar Z., Liu K.J.R. (2014). Time-reversal wireless paradigm for green Internet of Things: An overview. IEEE Internet Things J..

[5] Sabattini L., Levratti A., Venturi F., Amplo E., Fantuzzi C., Secchi C. Experimental comparison of 3D vision sensors for mobile robot localization for industrial application: Stereo-camera and RGB-D sensor. Proceedings of the 2012 IEEE International Conference on Control Automation Robotics & Vision (ICARCV).

[6] Cai X., Dean-Leon E., Somani N., Knoll A. 6D image-based visual serving for robot manipulators with uncalibrated stereo cameras. Proceedings of the 2014 IEEE/RSJ International Conference on Intelligent Robots and Systems (IROS).

[7] Hu X., Hu Y., Xu B. (2014). Generalized Kalman filter tracking with multiplicative measurement noise in a wireless sensor network. IET Signal Process..

[8] Wen Z., Cai Z. Mean shift algorithm and its application in tracking of objects. Proceedings of the 2006 International Conference on Machine Learning and Cybernetics.

[9] Jang W., Oh S., Kim G.A. hardware implementation of pyramidal KLT feature tracker for driving assistance systems. Proceedings of the 2009 IEEE International Conference on Intelligent Transportation Systems (ITSC '09).

[10] Karungaru S., Ishitani A., Shiraishi T., Fukumi M.A. (2012). simple interface for mobile robot equipped with single camera using motion stereo vision. Int. J. Mach. Learn. Comput..

[11] Sudin M.N., Nasrudin M.F., Abdullah S.N.H.S. Humanoid localization in a robot soccer competition using a single camera. Proceedings of the 2014 IEEE Tenth International Colloquium on Signal Processing & its Applications (CSPA).

[12] Using the Command Line for Communication with SSH and cURL. https://www.arduino.cc/en/Tutorial/LinuxCLI.

[13] A Wi-Fi Mobile Robot with Real-Time Video Transmission. https://youtu.be/8pajGYlh_UQ.

[14] Szeliski R. (2011). Computer Vision: Algorithms and Applications.

